# SKping cell cycle regulation: role of ubiquitin ligase SKP2 in hematological malignancies

**DOI:** 10.3389/fonc.2024.1288501

**Published:** 2024-03-15

**Authors:** Jonahunnatha Nesson George William, Ruby Dhar, Rohit Gundamaraju, Om Saswat Sahoo, Karthikeyan Pethusamy, A. F. P. Allwin Mabes Raj, Subbiah Ramasamy, Mohammed S. Alqahtani, Mohamed Abbas, Subhradip Karmakar

**Affiliations:** ^1^ Department of Medical, Oral and Biotechnological Sciences (DSMOB), Ageing Research Center and Translational Medicine-CeSI-MeT, “G. d’Annunzio” University Chieti-Pescara, Chieti, Italy; ^2^ Department of Biochemistry, All India Institute of Medical Sciences, New Delhi, India; ^3^ ER Stress and Intestinal Mucosal Biology Lab, School of Health Sciences, University of Tasmania, Launceston, TAS, Australia; ^4^ Department of Biotechnology, National Institute of Technology, Durgapur, India; ^5^ Institute of Environmental Protection and Sensors (IOS), Maribor, Slovenia; ^6^ Cardiac Metabolic Disease Laboratory, Department Of Biochemistry, School of Biological Sciences, Madurai Kamaraj University, Madurai, India; ^7^ Radiological Sciences Department, College of Applied Medical Sciences, King Khalid University, Abha, Saudi Arabia; ^8^ BioImaging Unit, Space Research Centre, University of Leicester, Leicester, United Kingdom; ^9^ Electrical Engineering Department, College of Engineering, King Khalid University, Abha, Saudi Arabia

**Keywords:** AML, leukemia, hematological cancers, miRNA, Skp2, oncogene, *in silico*, cancer genomics

## Abstract

SKP2 (S-phase kinase-associated protein 2) is a member of the F-box family of substrate-recognition subunits in the SCF ubiquitin-protein ligase complexes. It is associated with ubiquitin-mediated degradation in the mammalian cell cycle components and other target proteins involved in cell cycle progression, signal transduction, and transcription. Being an oncogene in solid tumors and hematological malignancies, it is frequently associated with drug resistance and poor disease outcomes. In the current review, we discussed the novel role of SKP2 in different hematological malignancies. Further, we performed a limited in-silico analysis to establish the involvement of SKP2 in a few publicly available cancer datasets. Interestingly, our study identified *Skp2* expression to be altered in a cancer-specific manner. While it was found to be overexpressed in several cancer types, few cancer showed a down-regulation in SKP2. Our review provides evidence for developing novel SKP2 inhibitors in hematological malignancies. We also investigated the effect of SKP2 status on survival and disease progression. In addition, the role of miRNA and its associated families in regulating *Skp2* expression was explored. Subsequently, we predicted common miRNAs against *Skp2* genes by using miRNA-predication tools. Finally, we discussed current approaches and future prospective approaches to target the *Skp2* gene by using different drugs and miRNA-based therapeutics applications in translational research.

## Introduction

Poly ubiquitination is the binding of numerous ubiquitin molecules into the same target protein. Generally, the polyubiquitination of proteins is induced by different signaling molecules and co-operates for protein degradation by the proteasomes. This post-translational modification process (Polyubiquitination) regulates numerous cellular events, including cell growth, proliferation, differentiation and apoptosis in mammalian cells (1). Any deregulation in the ubiquitination machinery and its components could disarrange the cellular homeostasis and initiate the process of neoplastic transformation in various cancers. The step by step action of the ubiquitin-activating (E1), ubiquitin-conjugating (E2), and ubiquitin-ligating (E3) enzymes associated with the ubiquitin-proteasome system (UPS), mediate ubiquitination by which they degrade targeted substrate proteins (2).

The SKP1, CUL1, F-box protein (SCF) complex consists of three core components that remain constant: RING-box 1 (RBX1), a RING-finger protein responsible for recruiting the E2 ubiquitin-conjugating enzyme; Cullin 1 (CUL1), acting as the scaffolding protein; and S-phase kinase-associated protein 1 (SKP1), an unchanging adaptor that links the core SCF complex with a variable F-box protein and its corresponding target protein (3). The specificity of the SCF complex for particular targets is determined by F-box proteins, with each F-box protein recognizing and binding a specific set of substrates. In humans, there are a total of 69 F-box proteins, categorized into three families based on their substrate recognition domains: (1) FBXW with WD40 repeats; (2) FBXL with leucine-rich repeats (e.g., FBXL1, also known as the S-phase kinase-associated protein 2 [SKP2]); and (3) FBXO with other domains (4). To regulate the levels of specific protein targets, each F-box protein recruits one of its substrates, often phosphorylated, to the core SCF complex, facilitating polyubiquitination and subsequent degradation by the 26S proteasome (5). With a total of 69 distinct F-box genes, it suggests the existence of up to 69 unique SCF complexes, each responsible for regulating a diverse array of protein targets (4).

Few well-characterized F-box proteins regulate substrates which are involved in cell cycle regulation, signal transduction, and transcription (**Table 1**) (33). Among these, one of the E3 ligases called SKP2 (S-Phase Kinase Associated Protein 2 (~ 45kDa)), a member of the F box family (34), is recognized as a pro-oncogene. These F-box proteins are mostly composed of one of the four subunits of ubiquitin-protein ligases complex named SCFs but do not always recognize substrates in a phosphorylation-dependent manner. In this complex of SCF’s, the F-box is referred to as a subunit, which serves as the recognition site for protein substrates. The N-terminal F-Box domain of the F-box binds to SKP1 and thereby connects with the SCF complex. After that, C-terminal Leucine-rich repeat (LRR) and WD40 repeats support substrate binding. Association of SKP1-SKP2 is found in humans (35). SKP2 assembles to SCF-type E3 ubiquitin ligase complex along with Cullin-1, Skp1, and Rbx1 (36–39). In addition, the requirement of cell cycle regulator CDK subunit 1 [CKS1] is important for CF SKP2-mediated ubiquitinylation of p27 (7).

**Table 1:** SKP2 and its known substrates.

Substrate	Function	Reference
E2A	B/T Cell Development	(6)
p27	Cell Cycle Control	(7)
p21	Cell Cycle Control	(8)
p57	Cell Cycle Control	(9)
p130	Cell Cycle Control	(9)
Cyclin D1	Cell Cycle Control	(10)
Cyclin E	Cell Cycle Control	(11)
Cyclin A	Cell Cycle Control	(12)
RAG2	DNA Repair	(13)
BRCA2	DNA Repair	(14)
ORC1P	DNA Replication	(15)
CDT1	DNA Replication	(16)
MKP1	ERK Signaling	(17)
TAL1	Erythroid Differentiation	(18)
E2F1	Gene Transcription	(19)
MEF	Gene Transcription	(20)
TOB1	Gene Transcription	(21)
MYC	Gene Transcription	(22)
MYB	Gene Transcription	(23)
FOXO1	Gene Transcription	(24)
FOXO3A	Gene Transcription	(24)
RBL2	Gene Transcription	(25)
MLL	Gene Transcription	(26)
UBP43	Interferon Signaling	(27)
USP18	Interferon Signaling	(28)
RASSF1A	Microtubule Stabilizer	(29)
SMAD4	Signal Transduction	(30)
CDK9	Transcriptional Elongation	(31)
HPV-E7	Viral Oncogenesis	(32)

**Figure 1:**
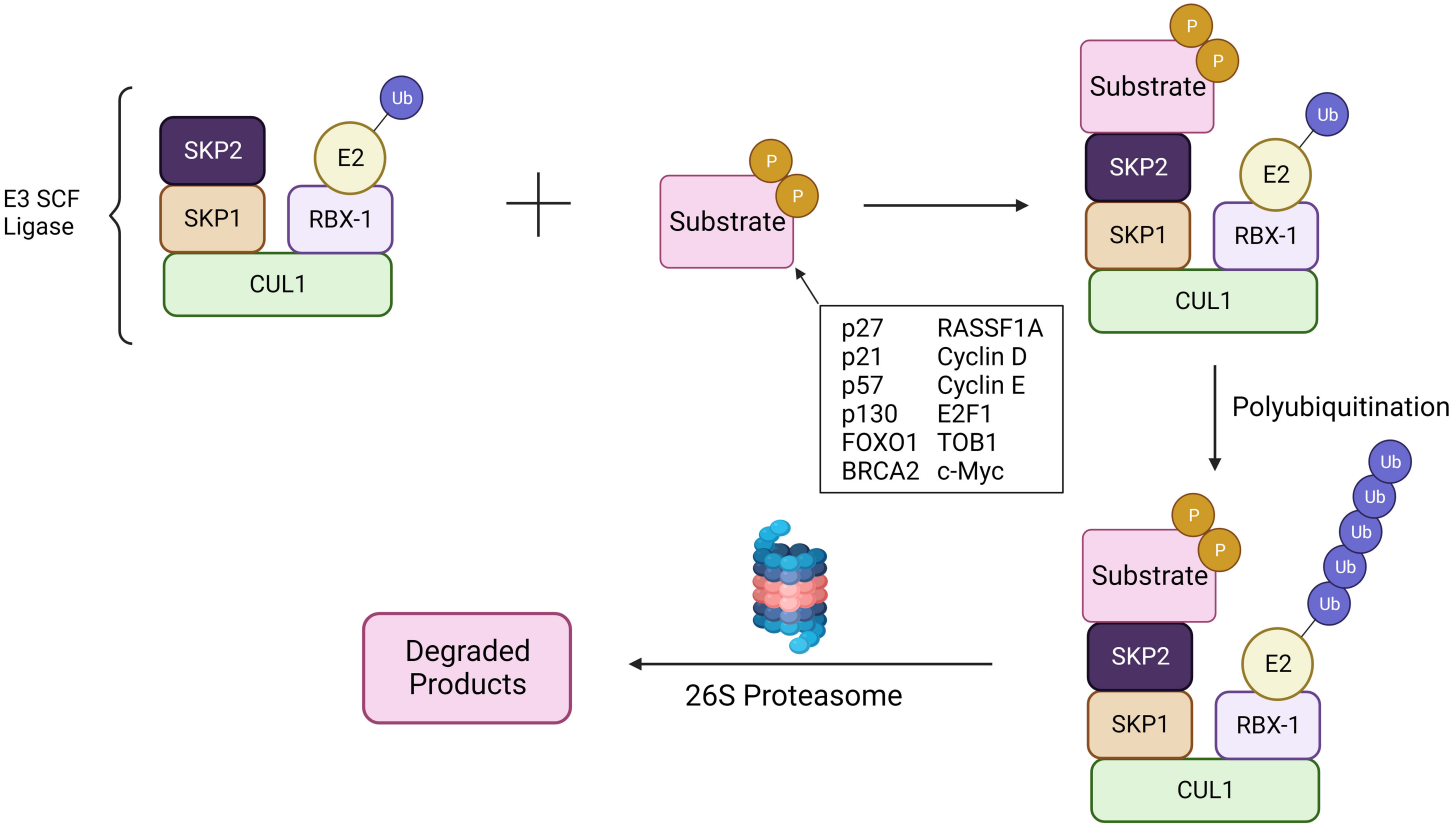
SCF^SKP2^ complex. The SCF^SKP2^ complex plays a pivotal role in regulating cell cycle progression and maintaining cellular homeostasis. SKP2, an F-box protein within the complex, acts as a substrate recognition component. It recognizes specific target substrates marking them for ubiquitination. Once ubiquitinated, the tagged proteins are targeted for degradation by the 26S proteasome. The SCF complex serves as an E3 ubiquitin ligase, facilitating the transfer of ubiquitin molecules to substrates. Ultimately, this polyubiquitination signals the proteasome to recognize and degrade the marked proteins, regulating key cellular processes and ensuring proper cell cycle dynamics.

*Skp2* gene plays a significant role in cell cycle progression and cell survival through ubiquitin-mediated degradation of many tumor suppressor proteins (p27, p21, p57, p130, FOXO1, BRCA2, RASSF1A, TOB1), cell cycle regulatory proteins (Cyclin D & E, E2F1, *etc.)* and oncogenes (*c-MYC, MYB*) (**Figure 1**) (40, 41). The target interruption of SKP2 leads to the accumulation of Cdk inhibitor p27, which leads to G1 phase cell cycle arrest. SKP2 mediates the degradation of p27 via ubiquitination through the 26S proteasome pathway (42, 43). Additionally, proteins like RING E3 ligases, are essential for the interaction of the E2-conjugating enzyme along with the SKP1adaptor protein (36). In addition, scaffold and ring finger proteins like Rbx1 are also required to target the substrate via its E3 ligase activity (36).

## SKP2 in cancers

**Figure 2:**
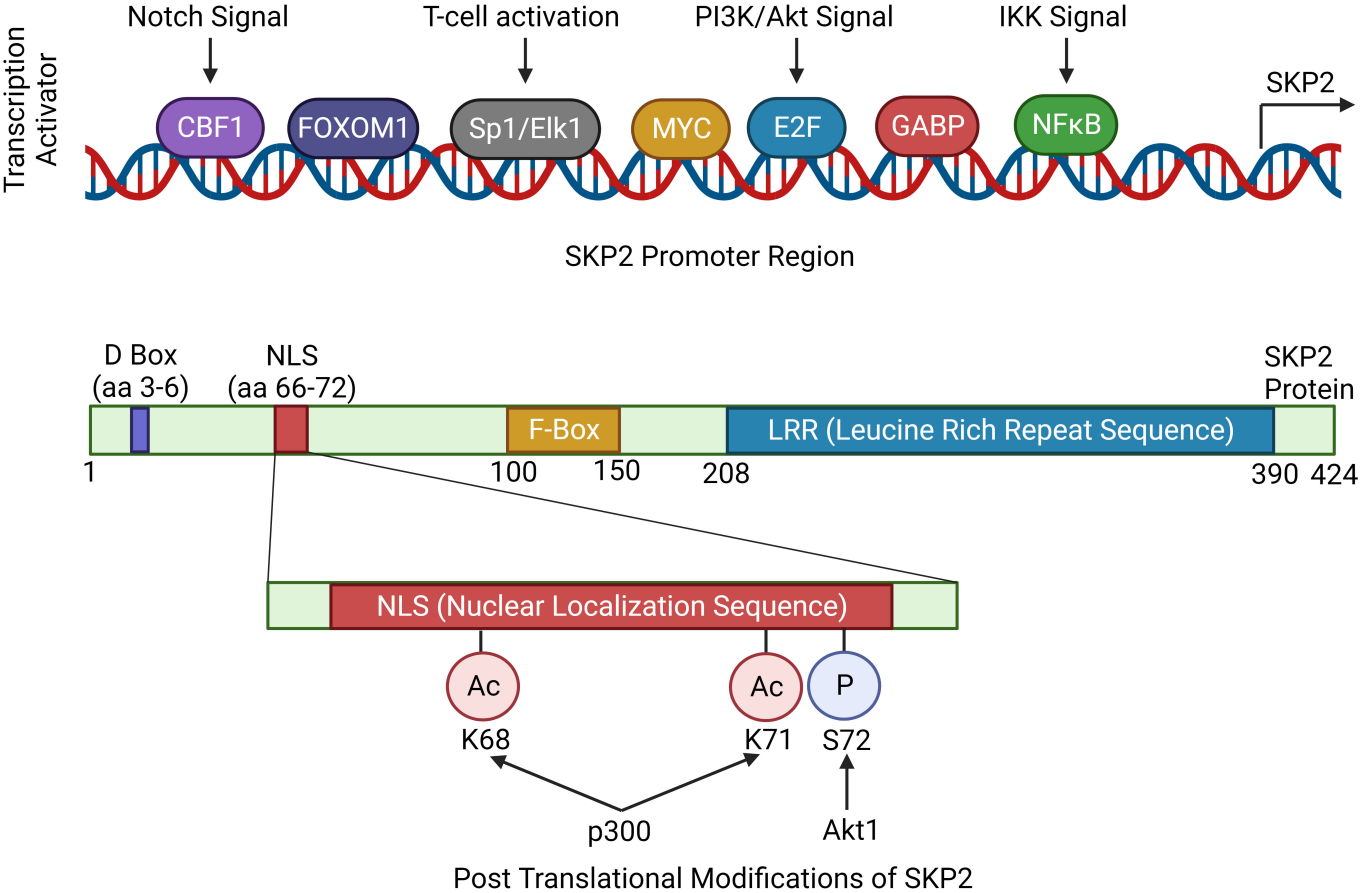
Regulation of *Skp2* gene expression. The expression of *Skp2* is intricately regulated by various signaling pathways, showcasing its significance in cellular homeostasis and proliferation. Key mitogenic signaling pathways like, Notch, PI3K/Akt and IKK, converges to SKP2 thereby modulating its expression. The coding region of *Skp2* contains functional domains essential for its function. The D-box is crucial for recognition by the anaphase-promoting complex (APC/C), marking SKP2 for degradation during cell cycle progression. The NLS (nuclear localization signal) guides SKP2 into the nucleus. The F-box domain is characteristic of SKP2’s role in the SCF complex, facilitating substrate recognition. Finally, the LRR (leucine-rich repeat) domain contributes to protein-protein interactions, enabling SKP2 to engage with other components of the SCF complex and its target substrates, orchestrating precise control over cell cycle checkpoints and cellular processes.

Higher expression of *Skp2* is associated with tumor initiation and progression (**Table 2**) (44). Concurrently, the level of SKP2 oscillates during the cell cycle and is controlled by both transcriptional and post-transcriptional mechanisms. During cell cycle regulation, low expression of *Skp2* is observed in both G0/G1 and late M/early G1, while a high level of SKP2 is found during G1/S transition, peaking at the S phase. Moreover, Cdk inhibitor p27 is usually stable in G0/G1 phase and unstable in the G1/S phase (8, 45). Cyclin E and E2F-1 proteolysis are essential for their rapid turnover during G1 to S phase progression, which directly increases the abundance of SKP2 during this time (46). Further, p300 acetylates SKP2 in the Nuclear Localization Signal (NLS) region, thereby mediates its localization in the cytoplasm, and enhances the stability of SKP2 (**Figure 2**) (43).

**Table 2:** SKP2 expression profile across tumor samples (derived from gepia2.cancer-pku.cn).

SKP2 Overexpressed Cancer	SKP2 Underexpressed Cancer
ACC – Adrenocortical Carcinoma	KICH – Kidney Chromophobe
BLCA – Bladder Urothelial Carcinoma	LAML – Acute Myeloid Leukemia
BRCA - Breast invasive carcinoma	PRAD – Prostate Adenocarcinoma
CESC - Cervical squamous cell carcinoma and endocervical adenocarcinoma	THCA – Thyroid Carcinoma
CHOL – Cholangiocarcinoma	
COAD - Colon adenocarcinoma	
DLBC - Diffuse Large B-cell Lymphoma	
ESCA - Esophageal carcinoma	
GBM - Glioblastoma multiforme	
HNSC - Head and Neck squamous cell carcinoma	
KIRC - Kidney renal clear cell carcinoma	
KIRP - Kidney renal papillary cell carcinoma	
LGG - Brain Lower Grade Glioma	
LIHC - Liver hepatocellular carcinoma	
LUAD - Lung adenocarcinoma	
LUSC - Lung squamous cell carcinoma	
OV - Ovarian serous cystadenocarcinoma	
PAAD - Pancreatic adenocarcinoma	
PCPG - Pheochromocytoma and Paraganglioma	
READ - Rectum adenocarcinoma	
SARC – Sarcoma	
SKCM - Skin Cutaneous Melanoma	
STAD - Stomach adenocarcinoma	
TGCT - Testicular Germ Cell Tumors	
THYM – Thymoma	
UCES - Uterine Corpus Endometrial Carcinoma	
UCS - Uterine Carcinosarcoma	

**Figure 3:**
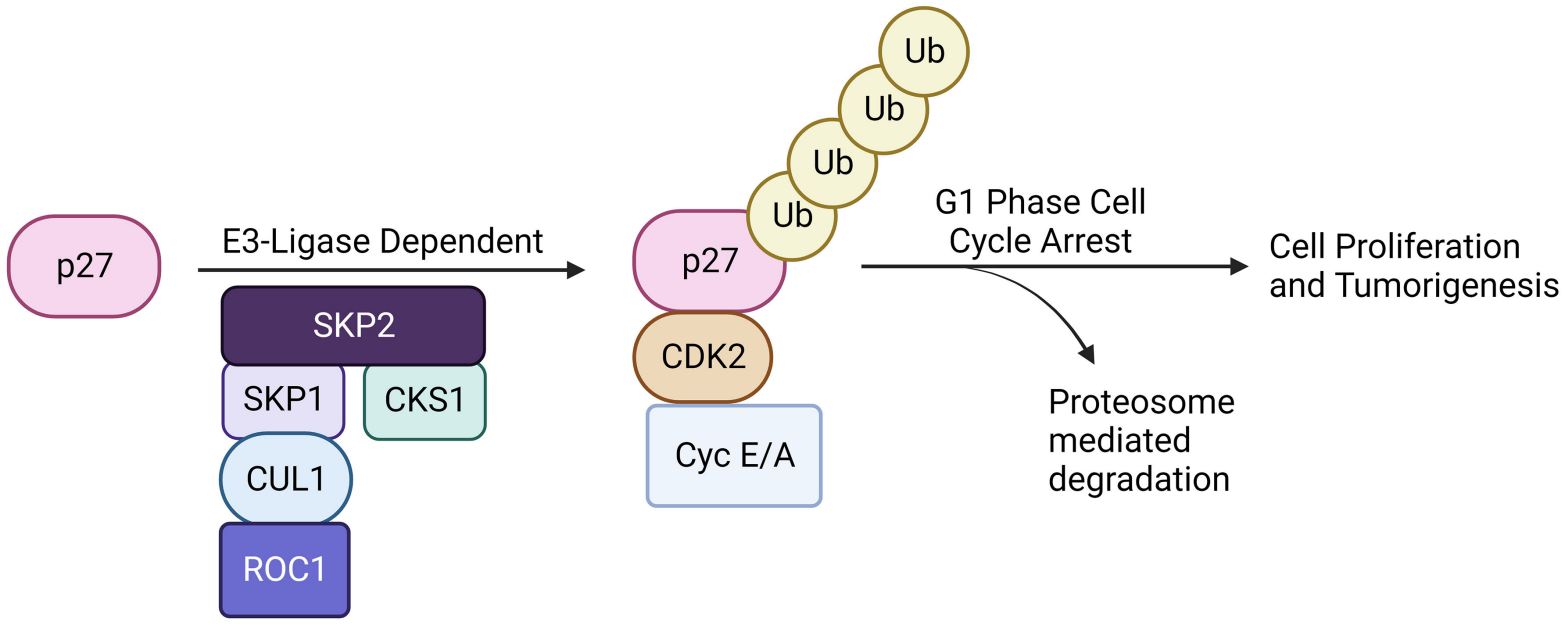
SKP2-p27 axis. The SKP2-p27 axis is a critical regulatory pathway governing cell cycle progression and proliferation. SKP2, a component of the SCF complex, targets the cyclin-dependent kinase inhibitor p27 for ubiquitination and subsequent proteasomal degradation. This ubiquitin-mediated destruction of p27 relieves its inhibitory effect on cell cycle progression, allowing cells to transition from G1 to S phase. Dysregulation of the SKP2-p27 axis is implicated in various cancers (solid and, including hematological malignancies), emphasizing its pivotal role in maintaining proper cell cycle control and highlighting its potential as a therapeutic targetin various malignancies.

A high level of *Skp2* and a low level of *p27* expressions are associated with poor prognosis in solid tumors. Similarly, an inverse correlation between *Skp2* and *p27* gene expression is also frequently found in hematological malignancies (47, 48). Thus an overexpression of the *Skp2* gene concomitantly decreases the expression level of the *p27* gene in diverse cancer types (**Figure 3**). However, the molecular mechanisms and the cause of *p27* gene loss and elevated levels of *Skp2* gene expression are not wholly investigated in all cancer types. To further support the significance of SKP2, an *in-vivo* xenograft mice model exhibiting high expression of the *Skp2* gene was found to promote tumor growth (46). Surprisingly, following depletion of the SKP2, tumor development is dramatically reduced by inducing programmed cell death and cell senescence (49). Furthermore, another study on glioblastoma cells also demonstrated that depletion of SKP2 inhibits cancer progression via promoting cellular senescence (50). Similarly, transgenic mouse models overexpressing *Skp2* have shown tumor growth in various tissues, but the cause of how SKP2 triggers neoplastic transformation is elusive (51).

**Figure 4:**
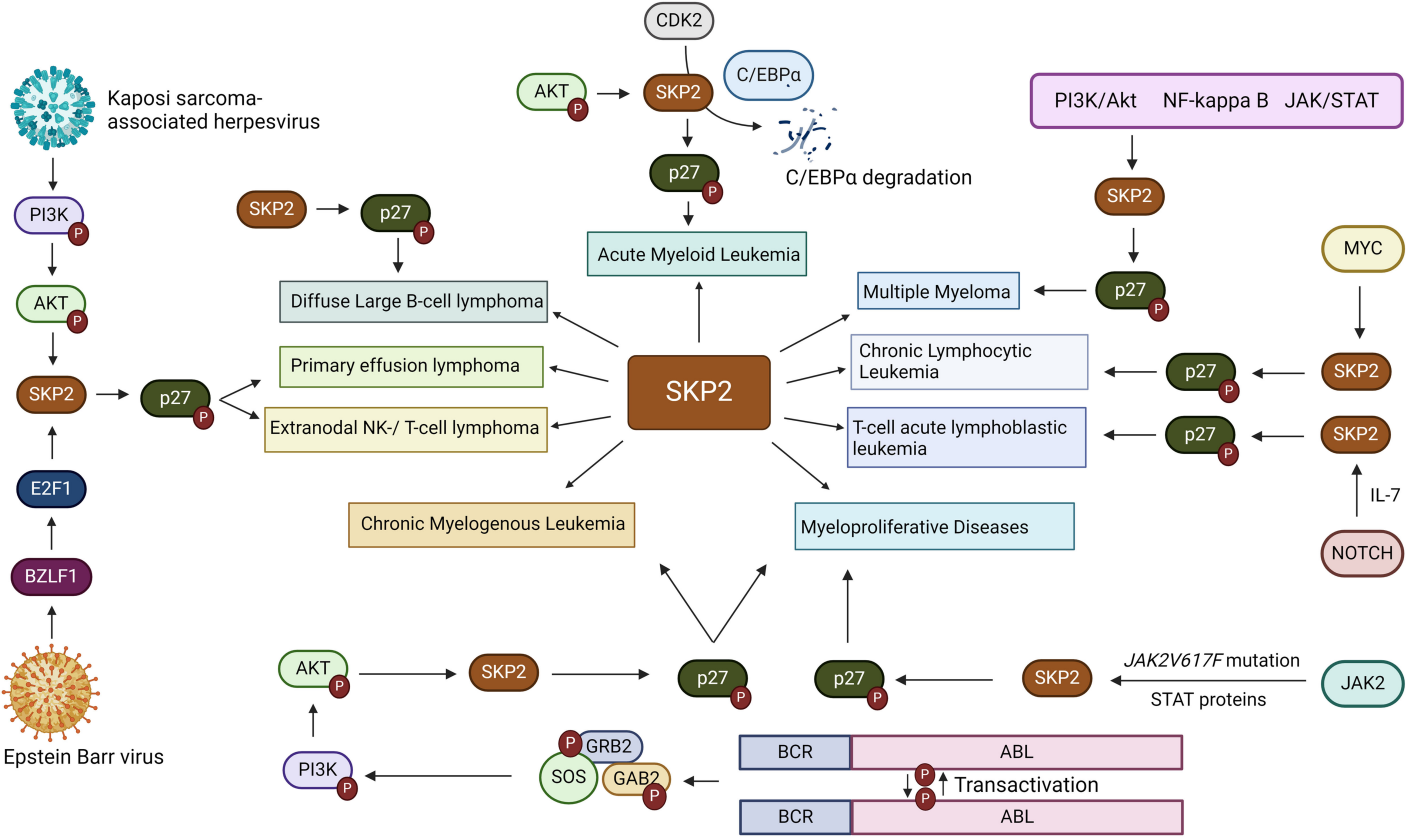
Involvement of SKP2 in hematological malignanices. SKP2, a critical player in hematological malignancies, features prominently in various cancers including acute myeloid leukemia (AML), chronic lymphocytic leukemia (CLL), and T-cell acute lymphoblastic leukemia (T-ALL), chronic myelogenous leukemia (CML), multiple myeloma, primary effusion lymphoma (PEL), and diffuse large B-cell lymphoma (DLBCL), Extranodal natural killer (NK)/T-cell lymphoma and myeloproliferative diseases. Mediated through diverse pathways such as PI3K/Akt, NFkB, and MYC, overexpression of SKP2 is often correlated with aggressive disease and poor outcomes, highlighting its significance in cancer biology and emphasizing the need for targeted therapeutic interventions.

While gene amplification may result in an enhanced *Skp2* expression in cancers, oncogenic signals could also contribute to its elevated expression. Oncogenic alterations leading to higher expression of *JAK2V617F* mutation, *BCR-ABL*, and *Her2/Neu*, which further activates Jak/Stat, and PI3K/AKT signals thereby inducing *Skp2* gene expression in malignant cells (33, 52). However, in the nucleus, BCR-ABL mediated transcription of *Skp2* is associated with PI3K/AKT/SP1 pathway and mTORC2 via mTOR signaling pathways, implicating the modulation of *p27* level expression. Mainly through PI3-kinase signaling, the mTORC2 pathway elevates the *Skp2* expression, thereby reducing the *p27* expression and initiating cancer progression (53, 54). Furthermore, p300 acetylates K68 and K71 residues of SKP2 during oncogenicity, sustaining their stability and enhancing retention in the cytoplasm (43). SKP2 promotes cellular invasion and migration by suppressing the tumor suppressor genes/protein expression and regulates its downstream targets, such as p21, p27Kip1, and FOXO1 (55). Aberrant regulation of the SKP2/p27 axis has also been noted in gastric cancer suppression, wherein MESP2 binds competitively to TCF4 (56). In addition, high SKP2 endorses cancer progression through the activation of various growth and survival-signaling pathways, for example, PTEN, ARF, pRB, FOXO1, and high Her2/Neu, *etc.* SKP2 acetylation and phosphorylation regulates its SCF E3 ligase activity in the cytoplasm during cancer progression, and AKT phosphorylates SKP2 at Ser72 during metastasis. The cytosolic SKP2 activates AKT and PTEN loss, implicating SKP2 translocation from the nucleus to the cytosol through Ser72 phosphorylation and induces tumor growth (57). Additionally, through neddylation, Cul-1 stabilizes the SKP2-SCF complex and negatively regulates the SKP2-SCF complex, Cul-1 dissociates from Cand1 by Cul1 neddylation and deneddylation of Cul1is mediated by Cop9-signalosome (CSN) protein complex (58). However, a complete SCF ligase activity is still largely unknown. Clinically, the elevated expression of *Skp2* is recognized as a poor prognostic marker in many solid tumor cancers and hematological malignancies (1). Concerning hematological malignancies, SKP2 being a crucial regulator of the cell cycle, plays a multifaceted role. SKP2 aberrations have been implicated in malignancies like acute myeloid leukemia, chronic lymphocytic leukemia, T-cell acute lymphoblastic leukemia, chronic myelogenous leukemia, multiple myeloma, primary effusion lymphoma, Diffuse large B-cell lymphoma, extranodal natural killer (NK)/T-cell lymphoma, myeloproliferative diseases etc. (**Figure 4**), disrupting hematopoietic differentiation and fostering genomic instability. The following delineates the role of SKP2 in the above mentioned malignancies.

## SKP2 in acute myeloid leukemia

*Skp2* expression is recognized as an independent prognostic factor in AML. High expression of *Skp2* is associated with shorter disease-free survival and overall survival. Interestingly, siRNA mediated knocking down of *Skp2* in AML cell lines HL-60/A resulted in cell cycle arrest reversing the multidrug resistance by downregulating *MRP* gene expression (59). However, further studies are required to showcase the cause of *Mrp* gene modulation in AML. The RNAi-based disruption of anti-miR-196b activity or pharmacologic inhibition of the Cks1-Skp2-containing SCF E3-ubiquitin ligase complexes significantly elevated the level of p27Kip1, which induces monocytic differentiation (60), noticeable reduction of leukemogenic potential, induced apoptosis and suppressing human AML growth (48). SKP2 and p27Kip1 are localized in the cytoplasm (61), which hints that an aberrant regulatory pathway is conducted through SKP2-mediated p27Kip1 proteolysis in most AML cases (62). On the other hand, SKP2 is positively correlated with phosphorylated PTEN, suggesting that the pPTEN-SKP2 axis might be a promising therapeutic target in AML (63).

AML is a complex heterogeneous disease with diverse pathologies. There are conflicting reports on the *Skp2* expression in AML. While results from TCGA shows a downregulation of SKP2 in AML (LAML), studies from other investigators reported an elevated SKP2 in AML (63). A possible reason for this apparent conflicting reports is due to the complex aetiology of AML. To improve the predictive value and therapeutic specificity of the *Skp2* gene in solid and hematological malignancies, we analyzed the TCGA data (data not shown). We performed different statistical analyses on diverse populations. Our TCGA analysis relied on an online portal exploration, and the data was generated with an interactive web-portal (UALCAN tools (http://ualcan.path.uab.edu) through a TCGA-level setup. The three different RNA-sequence gene expression data and 31 different clinical cancer types’ data were used for analysis, such as 1). Relative expression of the gene(s) across tumor and normal samples, as well as in various tumor sub-groups based on individual cancer stages over and under-expressed genes in individual cancer types, tumor grade, race, body weight, or other clinical pathologic features 2) effect of gene expression level on patient survival. Finally, we used 3) *in silico* validation studies for target genes (derived from the GENT2 database). Results depict that leukemia showed a marginally increased trend in *Skp2* expression as compared to lymphoma and Myeloma (**Supplementary Table 1**). Survival analysis also revealed a poor DFS (disease-free survival) with high *Skp2* expression, as also seen in leukemia vs lymphoma.

## Regulation of *Skp2* gene in other hematological malignancies

### SKP2 in Chronic Lymphocytic Leukemia (CLL)

Chronic Lymphocytic Leukemia (CLL) is the most commonly diagnosed leukemia in the Western world. CLL, also named B cell malignancy, is characterized by indolent lymph proliferative disorder, where immature B cells expressing CD5+, CD19, CD23, and CD20 B-cells progressively accumulate in the peripheral blood, bone marrow and lymph nodes (64). During the last decades, modern therapeutic approaches significantly improved to induce CLL apoptosis at various levels, but CLL remains incurable due to its drug resistance/relapse. Interestingly, the significantly higher expression [mean of 3 fold-protein] of a cell cycle inhibitor, p27, was detected in CLL tonsil and peripheral blood B lymphocyte samples as compared to healthy B cells. Besides, the expression of *Myc* is relatively low in CLL in comparison with normal healthy B cells. The inversely correlated MYC and p27 in CLL, and the larger set of CLL in cohort patient studies clearly demonstrated that the *Skp2* gene is involved in p27 degradation. In a similar report, high *Skp2* expression correlated with high *Myc* and low *p27* expression in most of the CLL cases. On the other hand, low SKP2 samples showed high p27, and the mean MYC protein levels were significantly higher than high SKP2 levels in comparison with Tonsil and CLL. These findings demonstrated that through the MYC-SKP2-p27 axis pathway, MYC induces p27 degradation via upregulating the *Skp2* gene in CLL (45).

### SKP2 in T-cell acute lymphoblastic leukemia

T-cell acute lymphoblastic leukemia malignancy is a subtype of leukemia arising from thymocytes. In fact, T-ALL constitutes around 12-15% of newly diagnosed cases of ALL in pediatric patients, notable for its distinctive clinical and biological characteristics (65). Based on the current modern combination therapy, long-term therapies are needed to be improved, especially with aged group patients. The molecular mechanism of different gene functions in T-ALL is complex, including the chromosomal translocation of *c-Myc, Hox 11, Tal1*, and *Lmo*, with the T-cell receptor locus (66, 67). High prevalence activation of mutated Notch signaling pathway emerged as an important genetic component for T-ALL pathogenesis. Interestingly, in T-ALL Notch, signaling pathways are found to regulate the *Skp2* expression and its protein target substrate p27. In T-ALL cells, the interaction of NOTCH 1 intracellular domain (ICD) with the *Skp2* promoter triggers *Skp2* expression levels and reduces p27Kip1 levels. The pharmacological agents blocking NOTCH signaling pathways reduce the expression of *SKP2*, and accumulate the p27Kip1, subsequently leading to G1 cell cycle arrest. Overall, NOTCH/SKP2/p27Kip1 axis might contribute to the pathogenesis of T-ALL (68).

### SKP2 in Chronic Myelogenous Leukemia

Chronic Myelogenous Leukemia (CML) is the type of leukemia cancer subtype where dysregulation of myeloid cell growth in the bone marrow leads to the accumulation of undifferentiated white blood cells in the blood. CML is characterized by the translocation of *BCR/ABL1* genes- chromosome t(9, 22)(q34;q11. 2). Almost 95% of CML patients have BCR/ABL translocation in the chromosomes (69). This translocation elevates the transcription level of SKP2 expression. SKP2-mediated p27Kip1 dysregulation has been observed in many types of cancers, and proteasome inhibitor BTZ reduces the expression of *Skp2* in CML (70). On the other hand, the inverse relationship between SKP2 and p27Kip1 has been noticed after the gene silencing of *Skp2* in CML (69, 71).

### SKP2 in multiple myeloma

Multiple myeloma (MM) malignancy develops due to uncontrolled plasma cell proliferation and relapses in most patients, which remains a challenge for modern chemotherapeutic treatments. Interestingly, Myristoylated alanine-rich C-kinase substrate (MARCKS) overexpression plays an essential role in drug resistance in MM. Activated MARCKS (p-MACKS) modulates the SKP2/p27-signaling axis. SKP2 mediates E2F1-induced cell proliferation and cell cycle progression through the reduction of p27Kip1. MARCKS activation by siRNA/drug (enzastaurin) reduces the MM resistance cell growth and induces apoptosis. The current study demonstrated that targeting MARCKS-mediated SKP2 will be a more helpful therapy against MM resistance. Furthermore, cyclin-dependent kinases regulatory subunit 1 (CKS1, encoded in humans by the *CKSB1* gene), cell cycle protein regulates p27Kip1, and p21CIP1 depends on *Skp2* expression. Similar to the above study, SKP2/p27Kip1, and CKSB1 were also found to be inversely correlated in MM cell lines (46).

### SKP2 in primary effusion lymphoma

Primary effusion lymphoma (PEL) is a rare, aggressive, immune-compromised type B cell lymphoma. It is associated with human herpesvirus type-8 infection, which commonly occurs in malignant effusions of the body cavities. In PEL, the *LANA-2* gene (KSHV latent gene vIRF-3), binds to SKP2 and regulates c-MYC-dependent gene transcription by recruiting *c-Myc*onin, its promoter regulatory region (72). Since *c-MYC* is a proto-oncogene, it regulates cell proliferation and survival in cancers. High expression of vIRF-3 induces the c-MYC ubiquitylation, plays a critical role in c-MYC mediated transcription, and stabilizes the c-MYC protein, leading to c-MYC-induced KSHV combined lymphomagenesis. Numerous studies have found that targeting SKP2 by proteasome inhibitor (MEG1320) or knocking down *Skp2*, stabilizes the p27Kip1, thereby triggering the mitochondrial-induced cell death by the caspase-dependent pathway (73). Interestingly, a plant compound Apigenin, also down-regulates the SKP2, stabilizes the p27Kip1 expression, and induces apoptosis in PEL cells (74).

### Diffuse large B-cell lymphoma (DLBCL)

Diffuse large B-cell lymphoma (DLBCL) is a sub-type of B-cell cancer. In adults, 30-40% of Non-Hodgkin’s Lymphomas are DLBCL, thereby portraying DLBCL as the most common type of Non-Hodgkin Lymphona (75). One of the biggest challenges of DLBCL is that there is a relapse recorded in more than 50% of patients succeeding treatment with increased mortality (76). The cause of DLBCL resistance is still unclear. However, numerous studies demonstrate that dysregulation of oncogenic/tumor suppressor gene regulation and impairment of repair pathways contribute to developing DLBCL relapse. Interestingly, SKP2 is highly observed in DLBCL, which is significantly correlated with the worst clinical outcome compared to low *SKP2*-expressing patients. Further, high *Skp2* in patients displayed a poor prognosis and less survival. High *Skp2* correlated with Ki-67 but not with p27, demonstrating SKP2 as an independent prognostic marker of clinical outcome (77). Bortezomib (BTZ) treatment reduces SKP2 via escalation of p27Kip1protein, including XIAP, cIAP1, and survivin, implicating the SKP2/p27Kip1 signaling pathway in DLBCL pathogenesis (78). Unfortunately, in other studies, Rituximab via the CHOP-mediated pathway did not provide beneficial outcomes for DLBCL patients with high *Skp2* and low *p27* expression (79).

### SKP2 in extranodal NK/T-cell lymphoma

Extranodal natural killer (NK)/T-cell lymphoma (ENKL) is a rare, aggressive type of malignancy in the lymph nodes besides GI tract, skin, and testis. ENKL shows poor survival among patients. The ENKT is often associated with Epstein–Barr virus (EBV) infection. The expression of *Skp2* levels is significantly increased, and an inverse correlation between SKP2 and p27Kip1 was observed with patients infected with EBV and phenotype of SKP+/p27– in ENKL (80). Overall these studies suggest that SKP2 plays a major role in the pathogenesis of ENKL carcinogenesis mediated through EBV (80, 81).

### Role of SKP2 in myeloproliferative diseases (MPD)

BCR-ABL induces MPD via impaired cell cycle regulation by destabilization of p27, which inhibits cyclin-dependent kinases (CDK). In contrast, BCR-ABL inhibition induces *p27* and reduces *Skp2*, which leads to G1 arrest (33). A similar regulation pattern was also observed where leukemic cells were transformed by FLT3-ITD, JAK2V617F, and TEL-PDGFRβ, which suggests that SKP2/p27 passage may act as a common target for leukemogenic tyrosine kinases. The *in vivo* mice transplanted with BCR-ABL–infected SKP2^_^/^_^marrow resulted in myeloproliferative syndrome with an increased survival rate compared with recipients of BCR-ABL-expressing SKP2-/- marrow (33). At the same time, in the SKP2-/^_^model, the nuclear *p27* expression is higher than SKP2-/-counterparts, demonstrating that leukemogenesis attenuation is regulated by high p27 levels in both MPD and CML (33, 82). The mutation of JAK2V617F commonly occurs in MPD, but in the case of its subset of polycythemia vera, homozygous JAK2V617F mutation is common. Therefore, mitotic recombination and duplication of the mutant allele are developed in MPD/CML. JAK2V617F mutation modulates the *Skp2* expression through STAT3/STAT5 transcription factors on the SKP2 promoter regulatory region (52). Therefore, inhibiting SCF-SKP2 for p27 stabilization recognition may be more beneficial for a therapeutic approach in MPD/CML and other hematological malignancies.

## Role of SKP2 in hematopoietic stem cells

The hematopoiesis process is a crucial step in producing diverse blood cells. This process undergoes long-term HSCs (LT-HSCs) and short-term HSCs (ST-HSCs), compartments that are the primary sources of hematopoiesis. LT-HSCs self-renewal themselves in order to maintain HSC pool and differentiate into multipotent progenitors, and they can further differentiate into lymphoid progenitors and myeloid progenitors, which produce mature blood cells, whereas ST-HSCs have limited self-renewal ability to differentiate into multipotent progenitors (69, 82). However, the mechanism of HSCs quiescence is largely unknown, and re-entering the cell cycle by HSCs is very crucial. Interestingly, SKP2 is involved in regulating HSC quiescence, pool size, self-renewal capability, etc. (83). In HSCs, the SKP2 deletion stabilizes the CKIs p21Cip1, p27Kip2, P57Kip2, and p130, increasing proliferation and reducing the stem cell self-renewal capability (83). SKP2 targets SKI inhibitors that inhibit cell cycle progression from G1 to S phase (68, 84). Due to myelosuppression and post-transplantation occurrences, high expression of *Skp2* is associated with neoplastic transformation, including HSC and its progenitors. High expression of *Skp2* sufficiently provides hematopoietic stress.

On the other hand, depletion of SKP2 reduces HSC mitotic activity and enhances HSC quiescence, increasing pool size and maintenance (83). The depleted SKP2 results in HSC impairment during myeloablative stress because of their inability to enter the cell cycle, thereby protecting HSC regeneration. SKP2 negatively regulates cyclin D1, which might be responsible for SKP2 maintenance of HSC quiescence, pool size, and self-renewal capability (9, 85, 86). SKP2 acts as a critical regulator for HSC quiescence and self-renewal capability and gives a novel paradigm for HSCs. SKP2 maintains the HSC homing and residence in the endosteal niche. SKP2 deficiency reduces the expression of b-catenin and its target genes. Since SKP2 maintains homing of HSC succeeding the post-transplantation, SKP2 might be helpful as a predictive marker for monitoring transplantation efficiency (87). Depleted *Skp2* expression enhances the sensitivity of HSCs and CMLs to chemotherapeutic drugs and triggers the long-term HSC reconstitution ability (9). Therefore, targeting SKP2 increases BM transplantation efficiency and sensitizes the cancer cell or CSC against chemotherapy. These model studies clearly demonstrate that future SKP2 targeting-based therapy will be an efficient approach against different cancer types, including HMs.

## Relationship between microRNAs and *Skp2* gene

MicroRNAs are small non-coding RNAs (10-24nts), and it regulate gene expression at the posttranscriptional level and play a critical role in cancer development (88). Mounting evidence displays the significant roles between miRNAs and *Skp2* gene expression. MicroRNA-186 regulates *Skp2* expression in pituitary tumors, induces p27Kip1-mediated cell cycle deregulation, and modulates cell proliferation. Similarly, human esophageal squamous carcinoma reduces cell proliferation and induces apoptosis (89, 90). In ovarian cancer, the expression of miR-30a-5p is low, but overexpression of miR-30a-5p reduces migration, invasion, and metastasis by posttranscriptional down-regulating *SKP2* gene expression (91). Since the miR-34a is downregulated in prostate cancer, overexpressing miR-34a downregulates RhoA and suppresses the c-Myc-SKP2 -Miz1 transcriptional assembly complex c-Myc-pTEFB complex that elongates transcription of numerous genes and affects the cellular function (92). Nevertheless, the reason for *Skp2* down-regulation through mir-34 has not been completely investigated yet in human renal carcinoma cells and prostate cancer (92). SKP2 mRNA is predicted to be a target of mir-7, but unfortunately, overexpressing miR-7 only reduces the SKP2 protein level but not at the transcriptional level. The SKP2-miR-7 mediated G1/S phase transition increases p27kip1 and reduces all G1 cell cycle indicators, such as Cks1, Cdk1/2, and CyclinD1/3, which suggests that overexpression of miR-7 arrests the CHO cell growth at G1 phase during cell undergoes stress (93, 94). miR-340 targets SKP2, inhibits non-small cell lung cancer tumor cell proliferation and induces apoptosis by targeting multiple negative regulators of p27 (95). Overexpressing miR-21-5p, miR-26-5p, and miR-30-5p in MCF-7 and tamoxifen-resistant MCF-7 cell lines showed marked reduction of SKP2 mRNA expression level (96). miR-203 targets SKP2 and regulates cell cycle and self-renewal in the hematopoietic stem cells and leukemia cells (97). Tumor suppressor miR-340 represses, the *Skp2* expression, inhibits tumor cell proliferation, migration, and invasion, and induces apoptosis in hepatocellular carcinoma (95). miR-26, miR-182, miR-340, and miR-506 share the 3’UTR of both SKP2 and PCNX and suppress their expression in non-small cell lung cancer (NSCLC) (98). Ectopic expression of miR-21 down-regulates the SKP2 in ovarian cancer cells (60). Apart from miRNAs, the long noncoding RNA meg3 and miR-3163 also coordinately repress the *Skp2* expression at the translation level and inhibit NSCLC cell growth, reducing NSCLC cell growth (99). miR-138 mimics or EZH2 inhibitor combined with a proteasome inhibitor, bortezomib-cavalcade, significantly reduces the MM tumors in a xenograft model by targeting RBPMS (100). To identify the SKP2 targeting miRNAs, we predicted through “TargetScan”, “MicroT-CDS” and “miRDB”: databases, where we found the seven most common miRNAs:` hsa-miR-21-5p, hsa-miR-590-5p, hsa-miR-26a-5p, hsa-miR-1297, hsa-miR-26b-5p, hsa-miR-30d-5p, hsa-miR-30a-5p (data not shown).

## Possible role of SKP2 on drug resistance in hematological malignancies–HM

In HM, the patients undergoing chemotherapy don’t respond to drugs. The molecular mechanism behind cancer/tumor cell’s resistance to chemotherapy is elusive. Surprisingly, overexpression of *Skp2* is associated with resistance and sensitization after pre-operative doxorubicin-based chemotherapeutically could aid in cancer cell death and successful chemotherapy in primary breast cancer patients (101, 102). SKP2 positively regulates the MAD2 via the p27-CDKs-E2F1 signaling pathway (103). Inhibition of SKP2 sensitizes paclitaxel-treated A549 and NCI-H1299 cells (103). SKP2 knockdown and/or inhibition sensitized the paclitaxel resistance prostate cancer cells, suggesting that SKP2 inhibitors might be the potential drugs against SKP2 upregulated cancers. Based on the SKP2 status in CML, USP10 inhibition significantly reduced the imatinib-sensitive and imatinib-resistant CML cell proliferation (71). Compound A (CpdA) interferes with SCF(SKP2) ligase by preventing the incorporation of SKP2 and induces G (1)/S cell-cycle arrest, SCF(SKP2)- and p27-dependent apoptosis, subsequently inducing p21 accumulation and other SCF(SKP2) substrates without affecting heat-shock protein response in MM (104). These studies indicate that SCF-SKP2 targeting agents may probably overcome the multidrug resistance mechanism and chemo-sensitize the MM cells (104). Furthermore, in breast cancer, SKP2 reactivates AKT-mediated resistance to PI3K inhibitors. Depletion of SKP2 reduces tumor growth in xenograft mice models (54). This study demonstrated that SKP2 plays a significant role in tumor progression and drug resistance. In lung cancer, small molecular inhibitors downregulated SKP2 and sensitized the lung cancer cells to paclitaxel. SKP2 has also been noted in stabilizing Mcl-1, conferring radioresistance in colorectal cancers (105).

In numerous malignancies, high SKP2 prevents apoptosis in a p53-dependent manner and promotes tumor progression and drug resistance (106). Combining SKP2 inhibitor C25 with bromocriptine sensitized the prolactinoma cells and induced apoptosis (107, 108). In multiple myeloma combinations of DT204, BTZ prevailed over drug resistance and induced apoptosis in proteasome inhibitors resistance cells. Both *in vitro* and *in vivo* model results strongly suggest that a combination of novel drug SCF-SKP2 inhibitor (DT204) and BTZ triggered synergistic anti-myeloma activity in the xenograft myeloma mouse model. Thus, targeting SCF-SKP2 by an SKP2 inhibitor combined with BTZ is a novel strategy to overcome drug resistance in MM. In addition, SKP2 inhibitor DT204 enhances the efficacy of BTZ-based therapies in multiple myeloma patients who are already BTZ-resistant (109). Proteosomal degradation of SKP2 also facilitates suppression of breast cancer growth by inducing autophagic cell death via F-box protein FBX041 (110). We previously showed that inhibition of pMARCKS potentiates BTZ-induced upregulation of p27 and p21 and downregulation of SKP2 (46). From a therapeutic perspective, it is noteworthy that targeting MARCKS can induce cell-cycle arrest and enhance apoptosis via E2F-1/SKP2/P27 axis in resistant MM cells (94). Therefore, identifying the molecular mechanism of drug resistance in HM is essential in the future. SCF-SKP2 inhibitors are the most widely used drugs to target the Ub++ proteasome system more precisely than PIs pharmacologically.

### Role of epigenetic modifiers in drug resistance

SKP2 is a key regulatory protein involved in controlling cell cycle progression and the degradation of specific target proteins (111). It plays a crucial role in maintaining normal cell growth and proliferation (111). However, dysregulation of SKP2 has been implicated in various cancers, including leukemia, and is also associated with drug resistance (109). Overexpression of *Skp2* in leukemia cells can contribute to drug resistance through several mechanisms (112). SKP2-mediated degradation of pro-apoptotic proteins may decrease the ability of cells to undergo apoptosis in response to chemotherapy (112). Enhanced cell cycle progression driven by SKP2 can lead to faster tumor cell growth, making it more challenging for drugs to keep pace with cell division (111, 113). SKP2 may influence DNA repair mechanisms, potentially reducing the effectiveness of DNA-damaging chemotherapeutic agents (114, 115). It is a critical player in regulating cell cycle progression and protein degradation, and its activity is intricately linked to epigenetic processes involving heritable changes in gene expression and chromatin structure without alterations in the DNA sequence (116). SKP2 can influence epigenetic regulation in multiple ways.

### Regulation of epigenetic modifiers

**Figure 5:**
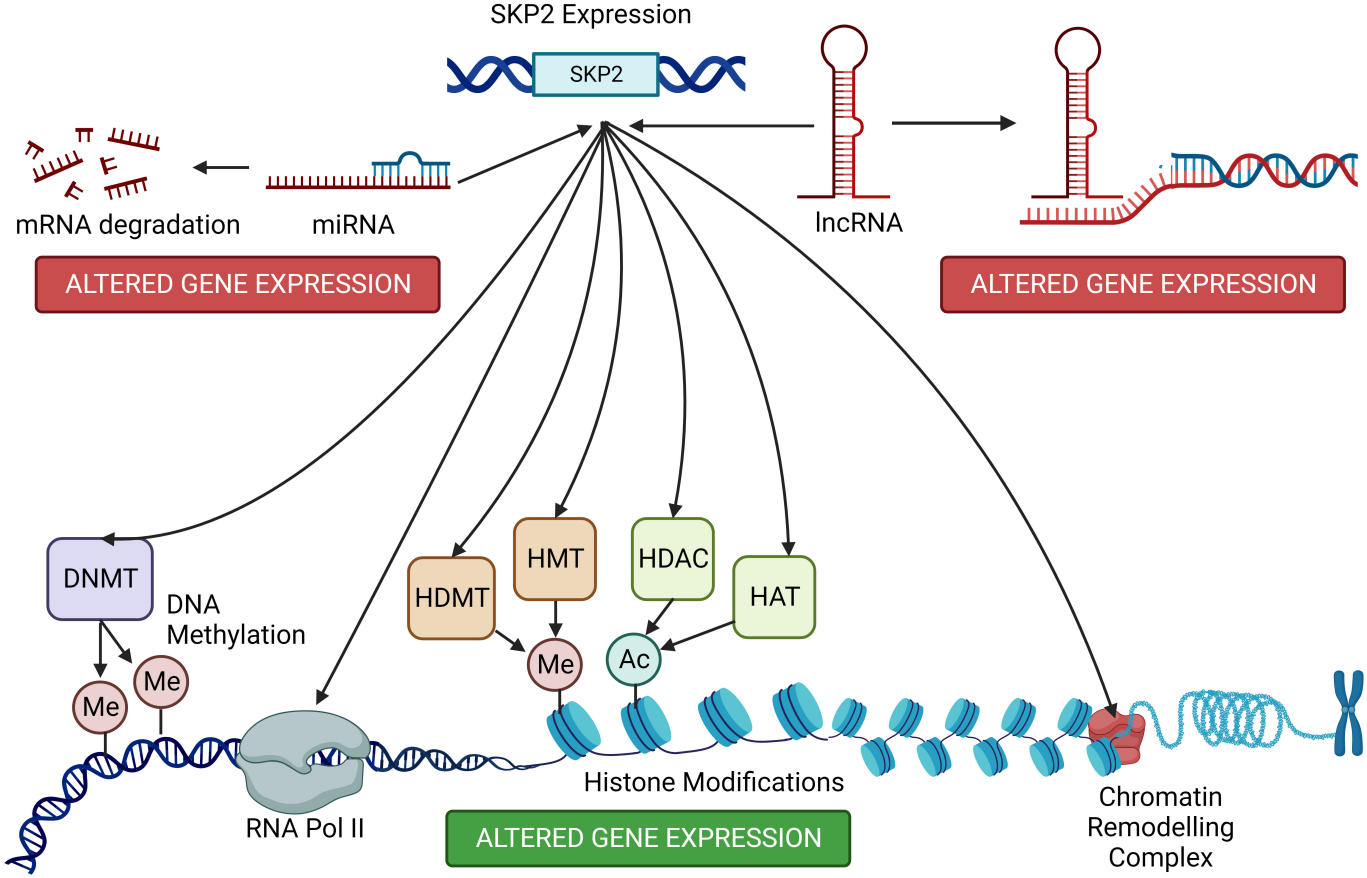
Host cell epigenetic modifications by SKP2. SKP2, beyond its canonical role in cell cycle regulation, influences host cell epigenetics through diverse mechanisms. Elevated SKP2 levels correlate with altered DNA methylation patterns and histone modifications, impacting gene expression. SKP2 promotes the degradation of key epigenetic regulators, disrupting the balance between chromatin modifications and transcriptional control. These modifications contribute to the development and progression of various diseases, including hematological malignancies. On the other hand, the expression of SKP2 is fine-tuned by a complex interplay of different miRNA and lnCNRA leading to its suppression or overexpression in different cancer types.

SKP2 can target specific proteins for ubiquitin-mediated degradation. Some of these target proteins include epigenetic modifiers such as histone deacetylases (HDACs) and histone methyltransferases (84, 117). By controlling the levels of these epigenetic modifiers, SKP2 can indirectly impact the acetylation and methylation status of histones, leading to changes in chromatin structure and gene expression (118). SKP2-mediated degradation of certain epigenetic regulators can affect chromatin remodeling complexes. Alterations in chromatin structure can lead to changes in gene accessibility, potentially impacting gene expression patterns. SKP2 can interact with various transcription factors and co-factors involved in epigenetic regulation (22, 119). These interactions can modulate the activity of transcription factors, influencing their ability to bind to specific genomic regions and regulate gene expression (**Figure 5**).

### Epigenetic effects on *Skp2* expression

Conversely, epigenetic modifications, such as DNA methylation and histone modifications, can also regulate the expression of *Skp2*. Aberrant epigenetic changes may result in dysregulated *Skp2* expression, contributing to altered cell cycle control and tumorigenesis. Epigenetic modifications can directly impact the expression of genes that are targets of SKP2-mediated degradation. Altered epigenetic regulation of these genes may influence their susceptibility to SKP2-dependent degradation (22). Dysregulation of *Skp2* and its interaction with epigenetic processes are associated with various diseases, including cancer (1). Aberrant *Skp2* expression and epigenetic alterations can contribute to tumorigenesis, metastasis, and drug resistance (1, 120, 121). Understanding the interplay between SKP2 and epigenetics is critical for unraveling the complexities of cancer biology and other diseases. Targeting SKP2 and its associated epigenetic processes may hold promise for developing novel therapeutic strategies, especially in the context of cancers where *Skp2* is dysregulated and contributes to disease progression. Additionally, research in this field continues to uncover the intricate mechanisms through which SKP2 and epigenetics intersect, providing insights into potential therapeutic targets and diagnostic markers. The regulation of SKP2 by epigenetic mechanisms plays a significant role in controlling its expression levels and activity. In the case of SKP2, several epigenetic mechanisms can in turn influence its expression.

The *Skp2* gene promoter is reported to be hypermethylated in some cancer types, decreasing *SKP2* expression (122). Reduced *Skp2* expression due to DNA methylation can contribute to cell cycle dysregulation and impact cancer progression (122). There is also a cancer-grade specific methylation. Results from TCGA depict that leukemia showed a marginally increased trend in *Skp2* expression compared to leukemia and myeloma. Survival analysis also revealed a poor DFS (disease-free survival) with high *Skp2* expression, as also seen in leukemia vs lymphoma (63, 123).

Histone modifications, including acetylation and methylation of histone proteins, can influence chromatin structure and gene accessibility. While histone H3 lysine 4 (H3K4) methylation is linked to gene activation, H3K9 and H3K27 methylation are associated with gene repression. Epigenetic changes in histone modifications near the *Skp2* gene are reported to modulate its transcriptional activity (124). In addition to histone modifications, specific miRNAs can target and degrade SKP2 mRNA or inhibit its translation, reducing SKP2 protein levels. Changes in miRNA expression profiles in cancer or other diseases can influence *Skp2* expression through post-transcriptional regulation as discussed before (125, 126). lncRNAs have been identified as regulators of *Skp2* expression, either by promoting its transcription or by destabilizing SKP2 mRNA (127).

Epigenetic changes can also influence the recruitment and activity of chromatin remodeling complexes that alter chromatin structure and gene accessibility. These complexes can either promote or inhibit the transcription of the *Skp2* gene by modulating the chromatin landscape around its promoter region. Epigenetic regulation of *Skp2* is particularly relevant in cancer, where dysregulated *Skp2* expression can contribute to uncontrolled cell proliferation and tumorigenesis (112). Understanding the epigenetic modifications that affect SKP2 and their functional consequences is essential for developing targeted therapies that can restore normal SKP2 regulation in cancer cells. Additionally, research in this area continues to uncover the intricate details of SKP2 epigenetic regulation and its implications in various diseases. SKP2 can also promote immune evasion in cancer by regulating immune checkpoint molecules, immune response pathways, Treg function, antigen presentation, and the overall immune microenvironment (127). Understanding the role of SKP2 in immune evasion is crucial for developing strategies to enhance immune responses against cancer cells and improve the efficacy of immunotherapies (1). Targeting SKP2 or its downstream signaling pathways may represent a potential approach to mitigate immune evasion and enhance the immune system’s ability to recognize and eliminate cancer cells.

## Future prospective of SKP2 inhibitors in HMs

**Figure 6:**
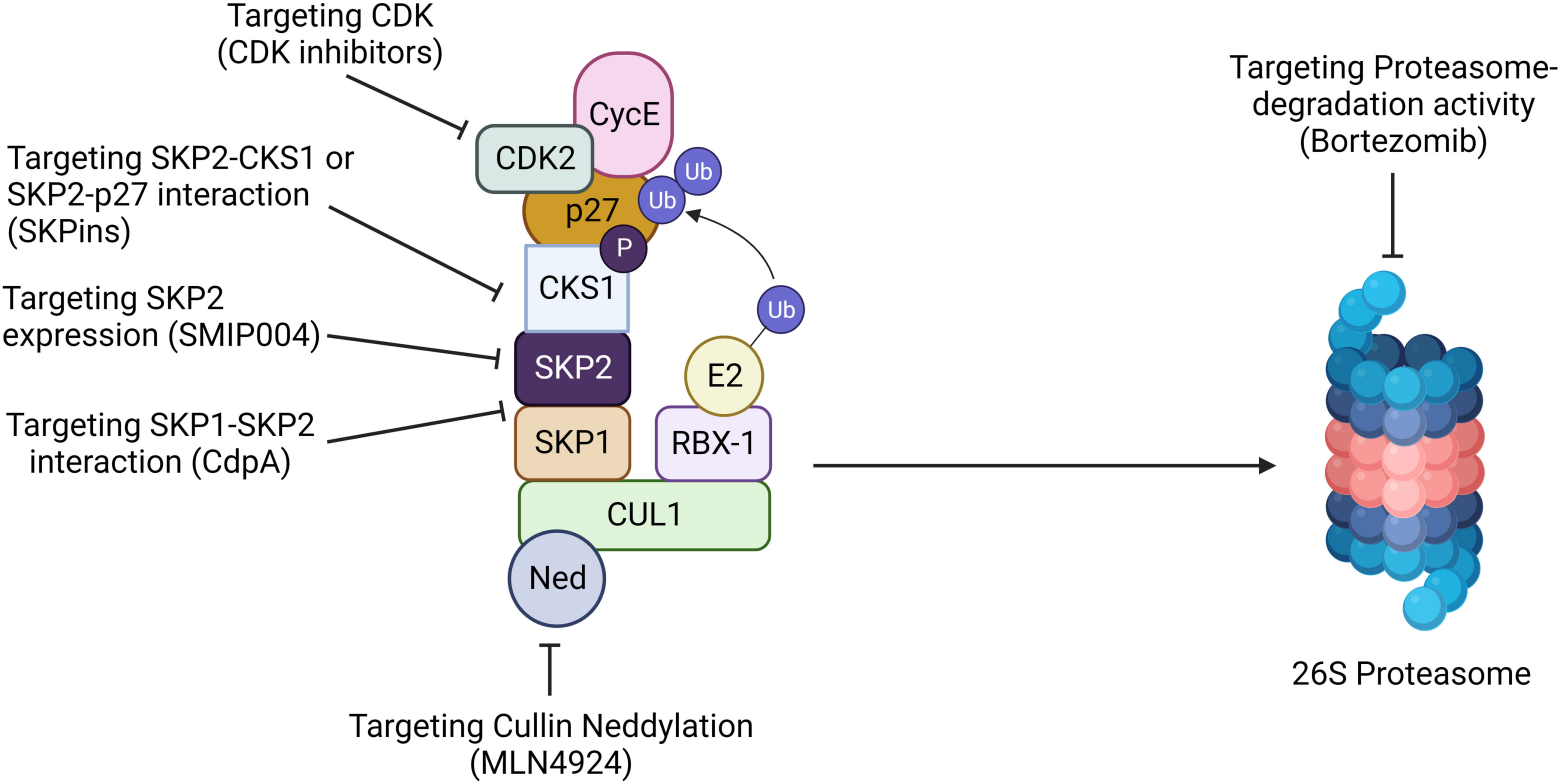
Targeting SKP2 for cancer therapy. Targeting SKP2 is emerging as a promising strategy for hematological malignancy. Inhibition of Cullin neddylation with MLN4924 disrupts SCF complex activity, leading to SKP2 degradation. CdpA hinders the SKP1-SKP2 interaction, while SMIP004 directly suppresses SKP2 expression. Compounds like SKPins disrupt SKP2-CKS1 or SKP2-p27 interactions, impeding cell cycle progression. Furthermore, targeting CDK inhibitors or using proteasome inhibitors like bortezomib prevents SKP2-mediated degradation of key proteins. These multifaceted approaches highlight the potential of SKP2 as a therapeutic target, offering diverse strategies to intervene in cancer progression and enhance treatment outcome.

Based on several reports, downregulation of *Skp2* induces the p27, promotes apoptosis, and sensitizes different types of cancers. However, further research is necessary to combat challenging tasks to identify the compound/inhibitors that are selectively employed for targeting the protein-protein interaction that holds the E3ligase together. Recently, the following inhibitors were developed against SKP2, named Bortezomib [FDA approved], Prodigiosin, Arsenic trioxide, Apigenin, curcumin, NSC689857, NSC681152, C1, C2, C16, C20, Compound A, Compound ZL25, and preclinical research compounds (**Figure 6**; **Table 3**) (8, 37, 74, 94, 109, 137, 162, 163).

**Table 3:** SKP2 inhibitors in hematological malignancies and solid tumors.

Tumor Type	Compound	Reference
Hematological Malignancies		
T cell Leukemia	SZL-P1-41	(128)
	SKPin C1	(128)
Myeloid Leukemia	Linichlorin A	(129)
	Diosmetin	(130)
	All-trans retinoic acid	(1)
Chronic Lymphocytic Leukemia	Bortezomib	(131)
Melanoma	Linichlorin A	(132)
	SKPin C1	(133)
	Betulinic Acid	(132)
	miR-590-5p	(134)
	Bortezomib	(135)
Multiple Myeloma	Neosetophomone B	(136)
	CdpA	(8)
	SKPin C1	(137)
	Bortezomib	(138)
Solid Malignancies		
Prostate Carcinoma	SZL-P1-41	(139)
	Gartanin	(140)
	Safranal	(141)
	SMIP004	(142)
	Flavokawain A	(143)
Lung Carcinoma	SZL-P1-41	(139)
	SKPin C1	(144)
	MLN4924	(144)
	Flavokawain A	(144)
	SMIP004	(103)
	Curcumin	(145)
	Tubocapsanolide A	(146)
Hepatocellular Carcinoma	Longikaurin A	(147)
	SZL-P1-41	(139)
Breast Carcinoma	Flavokawain A	(148)
	Linichlorin A	(149)
	Gentian Violet	(149)
	Diosgenin	(150)
	Rottlerin	(151)
	Curcumin	(24)
	Lycopene	(55)
	Quercetin	(55)
Osteosarcoma	SZL-P1-41	(139)
	Flavokawain A	(152)
Cervical Cancer	Linichlorin A	(149)
	Gentian Violet	(149)
Bladder Carcinoma	Flavokawain A	(143)
	ABT-751	(153)
Glioblastoma	Curcumin	(154)
Endometrial Carcinoma	SKP2E3Li C2	(155)
Colorectal Carcinoma	7-azaindoles	(156)
	Dioscin	(49)
	Sulforaphane	(157)
Pancreatic Carcinoma	Curcumin	(158)
	Rottlerin	(159)
Head and Neck Squamous Cell Carcinoma	Curcumin	(160)
Ovarian Carcinoma	Nitidine Chloride	(161)

BTZ, or pharmacological commercial name PS-341,/Valcade specifically, reversibly inhibits the 26S proteasome, an enzyme complex for regulating protein degradation under a controlled fashion. BTZ comprises of a peptide-like backbone and a boronate group, of which the latter exhibits a stronger binding affinity to the active site threonine, resulting in increased potency and selectivity toward the proteasome (164). In cancers, proteasomes’ inhibition leads to the building up of the protein substrates required for the cell cycle and apoptosis. Interestingly, BTZ suppresses the expression of *Skp2* and increases the *p27Kip1* expression in many types of cancers, and it has also been proven to significantly improve xenograft cancer cells in mice models. Furthermore, when BTZ combines with cisplatin, it suppresses cell proliferation and induces apoptosis by declining SKP2 and aiding in the accumulation of *p27* expression. Recent studies denote combining a novel SKP2 inhibitor DT204 and BTZ synergistically induced anti-myeloma activity and sensitized drug resistance in MM. The antiproliferative effect of BTZ in CML implies that proteasomal inhibitors are highly potent, thus suggesting that it might be beneficial for a strategic intervention for CML (109).

Considering natural products, Apigenin (4′, 5, 7-trihydroxyflavone) is a natural plant product commonly found in dietary flavonoids found in various fruit and vegetables. Hussain et al. demonstrated that Apigenin triggers apoptosis in Primary effusion lymphoma (PEL) cells, suppressing the activation of AKT/PKB pathway via downregulating *Skp2*, hypo-phosphorylation of Rb, and accumulating *p27Kip1* expression levels, which suggest that Apigenin may possibly have future therapeutic potential in PEL (74). Curcumin induces cell death by inhibiting PI3-Kinase/AKT Pathway in B-Precursor Acute Lymphoblastic Leukemia.

In addition, the pursuit of novel anti-HM therapeutic strategies does not just restrict to SKP2 inhibition, but also remains through selective inhibition of the ubiquitination–proteasome axis. To enhance specificity and selectivity in targeting, a promising avenue involves the focus on the E2–E3 complex (165, 166), given that the E2–E3 interaction imparts a high level of specificity and selectivity to the response by influencing specific ubiquitin bonds. Similarly, targeting the substrate binding domains of E3s offers a valuable opportunity. Inhibiting the interaction between a specific E3 and its target enhances specificity while minimizing off-target and side effects, potentially due to the limited impact on cellular events. As protein–protein interactions influence the specificity and selectivity of E3s, a deeper understanding of the three-dimensional structure of E3 enzymes through approaches like crystallography and cryo-electron microscopy will provide insights for developing novel inhibition strategies. Notably, Proteolysis Targeting Chimeras (PROTACs) (167) and molecular glues (168) represent effective approaches for promoting ubiquitination-mediated degradation of specific proteins, thereby contributing to increased substrate specificity.

Additionally, advancements in targeted drug delivery involve the use of cell membrane-coated nanoparticles (CNPs) and exosomes (169). CNPs, with their synthetic core containing anticancer drugs covered by a naturally derived cell membrane, offer a potential tool for precise delivery to disease sites. While promising, the translation of these strategies to clinical applications necessitates further technical improvements. Microenvironment-responsive drug-delivery systems based on nanoparticles and exosomes hold potential for guiding the release of SKP2/SCF component inhibitors in HM-specific microenvironments.

Moreover, the identification of synergistic combinations holds significant importance for advancing cancer treatment strategies. In the context of synthetic drug-target interaction, particularly those involving DNA damage-response genes and ubiquitination-mediated vulnerabilities, preclinical studies underscore the potential of combining these approaches with PARP inhibitors (170). CRISPR/Cas9- or RNAi- or shRNA-based whole genome screening, coupled with genomic and transcriptomic data analysis, contributes to predicting new synthetic lethal interactions for alternative anticancer therapeutic approaches. Recent discoveries, such as the identification of new substrates targeted by ubiquitination, including sugars alongside proteins, broaden the scope of this post-translational modification as a master regulator with potential implications for treating various pathologies, including HMs (171).

## Discussion and concluding remarks

HMs account for a substantial number of newly diagnosed cases in most oncology settings. These malignancies are most common in the Western world. They occur due to several factors, such as abnormality of cytogenetic, epigenetic, gene mutations, and other environmental factors that influence the progression of HM (172, 173). In HM, blood cancer cells grow uncontrolled and function abnormally. Commonly, HM’s are categorized into three major subtypes: such as Lymphoma, Leukemia, and Myeloma (174). Among these, AML is developed among adults. More than 80% of cases re-occurred in patients over the age of 60, and 20-30% of cases are children. However, the average survival rate among all HM types is small, especially among elderly patients due to drug-resistant drug resistance or relapse after advanced chemotherapy and transplantation treatments. The molecular mechanism of drug resistance or relapse is not entirely understood in HMs. Compared to proteasomal inhibitors BTZ, E3 ligase drugs specifically block the entire protein degradation with less toxicity.

The components of E3 ligases such as MDM2, FBW7, RBX2/ROC2, RBX1/ROC1, Cullins, and many others are referred to as oncogenes or tumor suppressors; similarly, essential proteins such as p53 and Notch are associated during cancer development. The inverse correlation between SKP2 and p27 cell cycle regulators in HM and solid cancer demonstrated the shared mechanism of neoplastic transformation (175). However, *Skp2* gene function has not been fully investigated in hematological malignancies. Hence, we analyzed in-silico RNA seq using available TCGA datasets. Our results (data not shown) demonstrated that the M7 AML within the French American classifications exhibited a high expression of *Skp2*, while there seems to be no significant difference in expression among different ethnic races. High *Skp2* gene promoter methylation among African American populations illustrates the complexity of epigenetic aberration (data not shown), and increased expression of *Skp2* across tumors demonstrates a common mechanism of SKP2 drug resistance. Poor survival rates among the African American population and FLT3 mutation suggested that common mutation patterns are linked with overexpression of the *Skp2* gene (figure not shown). Our heatmap analysis demonstrated both positively and negatively correlated genes with SKP2; identifying the relationship with complex gene network functions that may support drug resistance mechanisms in various aspects (**Supplementary Table 2**). In addition, our STRING analysis (data not shown) demonstrated protein networks of SKP2 interaction, which will prompt the identification of the associated factors involved in the drug resistance mechanisms. Escalated expression of *Skp2* in Basso Lymphoma results demonstrated strong evidence of a common mechanism involved in triggering the *Skp2* gene in HM and a crucial player in Hematopoietic stem cells (data not shown).

Similarly, miRNAs are also found to be a crucial player in drug resistance affecting various genes. Hence, we highlighted the *SKP2* gene function by projecting the essential role of miRNAs across various types of solid tumor malignancies. However in HM, only miR-203 was found to target SKP2 in Leukemia and hematopoietic stem cells.

Our miR target prediction and Venn diagram analysis demonstrated common miRNAs such as hsa-miR-21-5p, hsa-miR-590-5p, hsa-miR-26a-5p, hsa-miR-1297, hsa-miR-26b-5p, hsa-miR-30d-5p, hsa-miR-30a-5p *Skp2* gene (data not shown). Overexpression of mir-21-5p induces apoptosis and cell cycle arrest by down-regulating SKP2 and overcoming Bortezomib resistance in Multiple Myeloma. Similarly, some reports demonstrated that targeting the EZH2/miR-138 axis might be a potential therapeutic target against MM (100). Nevertheless, more evidence is required to validate *Skp2* gene regulation and its function in other HM, including AML (100). In the future, identifying the posttranscriptional and feedback regulatory loop mechanism of SKP2 will support the miRNA rational therapeutic approaches against relapse. Finally, we demonstrated the significance of SKP2 targeting drugs in HM, which strongly suggests a potential therapeutic strategy; however, miRNA mimics/miRNA inhibitors alongside natural products combination such as Apigenin, Dioscin, Arsenic trioxide, NSC689857, NSC681152, C1, C2 with, C16, C20, Compound A, and Compound ZL25 will open a new doorway for understanding the molecular mechanism of drug resistance or relapse in HM patients (49, 74, 160, 163, 176).

SKP2 remarkably promotes phosphorylation, ubiquitination, and degradation of PDCD4 (Programmed cell death protein 4), thereby facilitating cell proliferation and survival in breast cancer cells. SKP2 and PDCD4 displayed an inverse correlation in this cancer (177). Interestingly, its expression exhibits dynamic patterns, with some cases demonstrating overexpression in cancer samples compared to normal tissues, while others exhibit elevated levels in control samples relative to cancerous tissues. A high throughput screening identified SKP2 as a potentially novel cancer drug target (41), suggesting that pharmacologic SKP2 inactivation may limit tumor progression and overcome chemoresistance. However, in prostate cancer, SKP2 exhibits an opposite trend with high expression associated with a gain in mesenchymal and CSC-like phenotype compared with epithelial cells (178). This variability underscores the complexity of SKP2’s role in cancer progression and highlights the need for personalized therapeutic approaches. Given its diverse implications, personalized therapies targeting SKP2 may offer a tailored strategy to address the specific expression patterns observed in individual patients, potentially enhancing treatment efficacy and minimizing adverse effects. As researchers unravel the intricacies of SKP2’s involvement in hematological malignancies, the exploration of targeted interventions holds promise for advancing precision medicine in cancer therapy.

Thus, we can conclude that SKP2 is critical in regulating multiple cellular functions related to cell growth, differentiation, and cell cycle. These alterations could perturb the delicate balance and contribute to different pathological states like cancer. But the in-depth and detailed exploration of these aspects of SKP2 biology will be not only helpful in understanding cancer but also in discovering a therapeutic target. It has been observed that SKP2 dysregulation is one of the fundamental driver events for oncogenesis. These observations show that SKP2 is an oncogenic modulator; hence, its expression status is vital in cancer prognosis and determining treatment response. Small molecule activators or inhibitors for SKP2 hold tremendous promise against various cancers. It was reported that an expression of *Skp2* confers drug resistance, and hence targeting SKP2 appears to be crucial for overcoming drug resistance in cancer chemotherapy. Therefore efforts have been made to develop novel inhibitors targeting SKP2 (43). However, more clinically relevant human tumor models, such as PDX and organoids and genetic mouse models should be applied to carefully evaluate the efficacy of Skp2 inhibitors. Further research is of utmost necessity to delineate the signaling pathway for SKP2 and identify its cellular target function to understand the molecular mechanism of drug resistance in different cancer types.

## Author contributions

JW: Writing – original draft. RD: Data curation, Writing – review & editing. RG: Writing – review & editing. OS: Writing – review & editing. KP: Methodology, Writing – review & editing. AR: Methodology, Writing – review & editing. SR: Methodology, Writing – review & editing. MSA: Data curation, Funding acquisition, Writing – review & editing. MA: Data curation, Funding acquisition, Writing – review & editing. SK: Conceptualization, Project administration, Writing – original draft.

## Supplementary material

Supplementary Table 1 SKP2 expression in Lymphoma, Leukemia and Myeloma.

Supplementary Table 2 Positive and Negatively correlated genes with SKP2.
